# Simultaneous Evaluation of Pulse Contour Devices Using an Innovative Hemodynamic Simulation Bench

**DOI:** 10.3390/jcm14228030

**Published:** 2025-11-12

**Authors:** Paul Samuel Abraham, Bernardo Bollen Pinto, Raphael Giraud, Thomas Millien, Sylvain Thuaudet, Karim Bendjelid

**Affiliations:** 1 Anaesthesiology Department, Lausanne University Hospital, 1011 Lausanne, Switzerland; 2 Faculty of Medicine, University of Geneva, 1211 Geneva, Switzerland; 3 Department of Anaesthesiology, Pharmacology, Intensive Care and Emergency Medicine, Geneva University Hospital, 1211 Geneva, Switzerland; 4 S.T. Consulting, 14480 Le Fresne-Camilly, France

**Keywords:** hemodynamic monitoring, pulse contour analysis

## Abstract

**Introduction:** Evaluating cardiovascular function is crucial in the care of critically ill patients. Recent advancements in continuous cardiac output (CO) monitoring have led to the emergence of several arterial pulse contour devices. To effectively compare the accuracy of these devices, a comprehensive assessment is necessary. However, no experimental studies were found that have evaluated these devices in a controlled setting. **Methods:** In this innovative bench study, we used a Donovan mock circulatory system in conjunction with a total artificial heart (TAH-t) to simultaneously generate several comparable arterial waveforms and compared CO estimates from three different pulse contour devices: FloTrac™ (Vigileo™, v1.8 4th generation, Edwards LifeSciences, Irvine, CA, USA), proAQT™ (PulsioFlex™, Pulsion Medical Systems, Munich, Germany), and LiDCO™ Plus (LiDCO™, LidCO Ltd., Cambridge, UK). These devices underwent several hemodynamic challenges (HCs), including decreased preload, decreased afterload, and increased heart rate. To evaluate the degree of agreement between the devices, we performed a Bland–Altman analysis for the paired devices. The interclass comparison, error percentage, and variation coefficient for each device were also assessed. **Results:** The present study first tested the comparability between the three additional arterial line waveforms, and the arterial control line was simultaneously generated with the hemodynamic simulation bench. Comparing the reference values of the dP/dt and sAUC pulse pressure, we found no clinically significant difference between the simultaneously generated arterial waveforms. The different pulse contour devices were then each connected to the arterial lines, with the performance of HCs. HC1 with a decreased preload revealed that CO estimates significantly decreased compared to the baseline values: 3.2 ± 0.06 L.min^−1^, 4.7 ± 0.05, 4.3 ± 0.07, and 4.0 ± 0.05 for reference methods FloTrac™, PulsioFlex™, and LiDCO™, respectively. HC2 with an increased heart rate revealed CO estimates with FloTrac™, PulsioFlex™, and LiDCO™—6.0 ± 0.03, 6.6 ± 0.06, and 6.0 ± 0.05 L.min^−1^, respectively—when the CO estimate was 5.6 ± 0.2. HC3 with a decreased afterload that significantly increased CO estimates compared to the baseline with FloTrac™, PulsioFlex™, and LiDCO™—7.0 ± 0.18, 6.6 ± 0.15, and 7.1 ± 0.30 L.min^−1^, respectively—when the CO estimate with the reference method did not change significantly (from 5.90 ± 0.13 to 5.94 ± 0.11 *p* = 0.26). The devices’ degree of agreement was estimated with Bland–Altman analysis. **Conclusions:** The Donovan Mock Circulatory System with SynCardia TAH-t can be used as an innovative experimental hemodynamic simulation bench. It was proven to be stable, accurate, and reliable in generating several controlled pulse pressure waveforms, while many parameters could be changed, such as the preload, heart rate, or afterload. This enables a simultaneous evaluation of different pulse contour devices submitted to several HCs. This is of interest for clinicians to better understand the underlying principles and realistically compare the performance and potentially inherent limitations of pulse contour devices experimentally in a controlled simulated environment.

## 1. Introduction

Evaluating cardiovascular function is a crucial part of caring for critically ill patients or high-risk patients under anesthesia. Pulmonary artery catheters were the first widely available devices used to easily monitor cardiac output (CO) at the bedside [1,2]. Recent advancements in continuous cardiac output (CO) monitoring have led to the emergence of several arterial pulse contour devices [3]. Each device consists of a specialized software algorithm, most of which are based on the findings of Otto Frank [4]. While there may be a positive statistical correlation between pulse contour and stroke volume, it is not significant enough that one may be predicted from the other with high accuracy. Such a prediction must depend on the evaluation of individual arterial distensibility, knowledge of compliance in the arterial tree and its several parts, and the estimation of arteriolar drainage [5]. Pulse contour devices are generally classified as either calibrated or non-calibrated. Some pulse contour devices can be used with standard disposable pressure transducers (LiDCO™) when others may require the use of specific disposable pressure transducers (FloTrac/Vigileo™ and The ProAQT/Pulsio-Flex™) responsible for an increase in carbon dioxide emission and additional cost [6]. If various devices allowing for continuous cardiac output measurement are now commercially available, a variety of factors may influence the selection of a cardiac output monitoring device (institutional predicted cost, device accuracy and performance, ease of use, invasiveness) [7].

Despite the growing availability of minimally invasive technologies, a significant gap remains in perioperative hemodynamic monitoring research. Arterial pulse waveform devices and their underlying algorithms differ, and the heterogeneity of existing validation studies hinders their direct comparison with each other. Most of these studies are conducted under uncontrolled clinical conditions, where patient variability, pharmacologic interventions, and dynamic physiological changes introduce substantial bias. To enable meaningful evaluation of device performance and limitations, controlled experimental models are needed to allow for the simultaneous, head-to-head assessment of multiple pulse contour systems under standardized and reproducible conditions [8]. Such an approach would provide a more objective comparison of device accuracy and reliability, thereby improving clinical decision-making in perioperative cardiovascular management.

Clinical studies simultaneously comparing several pulse contour algorithms to a reference cardiac output measurement technique are scarce [9]. Some algorithms have been shown to underestimate CO in the context of vasodilation and vasopressor administration [10], while others appear unaffected [11]. These findings emphasize the need for the experimental testing of pulse contour algorithms to effectively compare the accuracy, agreement, and performance among existing pulse contour devices. No studies were found to have evaluated these pulse contour devices using an experimental bench and an accurate reference method.

The purpose of the present study is the use of an innovative hemodynamic simulation bench with a Donovan Mock Circulatory System (DMCS) associated with a total artificial heart (TAH-t) to generate several comparable and simultaneous arterial lines under controlled settings, which are then submitted to pulse contour devices to evaluate their performance. Three different pulse contour devices were evaluated in this study: proAQT™ (PulsioFlex™ Pulsion Ltd., Munich, Germany), LiDCO™ Plus, (LiDCO™, LidCO Ltd., Cambridge, UK), and FloTrac™ (Vigileo™, v 1.8, 4th generation, Edwards LifeSciences, Irvine, CA, USA). All these devices were tested with major changes in preload, heart rate, and afterload, described later as hemodynamic challenges (HCs).

## 2. Materials and Methods

### 2.1. The Bench Description

Our innovative hemodynamic simulation bench was developed with a DMCS associated with TAH-t (SynCardia™ Systems, Inc., Tucson, AZ, USA). See the Supplementary Material for pictures (Figure S1), videos (Films 1 & 2), and a description of the mock pulsatile circulatory system.

### 2.2. The Bench Settings

The baseline settings for the SynCardia driver were adjusted as follows: 110 beats per minute with 40% systole; left driving pressure, 160 mmHg; right driving pressure, 60 mmHg; and left and right vacuum, −6 mmHg. The SVR and PVR reservoirs were placed 20 cm and 10 cm high, respectively, from the upper side of the bellows (see the Supplementary Material).

Pressure in all 4 tanks of the DMCS was continuously measured and displayed in real time on an adjacent screen (PowerLab^®^ pro 8, AdInstrument, Sydney, Australia). Each chamber disposed of a pressure line that allowed the monitoring of the maximum, minimum, and mean pressure in every tank (mmHg). Therefore, a pressure transducer connected to these chambers provided artificial right atrial pressure (RAP), pulmonary artery pressure (PAP), left atrial pressure (LAP), and arterial pressure in mmHg. The observed values were similar to the physiological conditions of the chosen dual-drive console settings described earlier. After calibrating and zeroing, the pulsatile signal quality enabled observation of systolic and diastolic pressure, with dicrotic notch. The quality of the arterial signal through the fluid-filled catheter associated with the flushing bag pressure at 300 mmHg was controlled with a fast flushing square wave test before every data recording to exclude an over- or under-damped arterial line waveform [12].

### 2.3. Bench CO References Methods

These bench settings generated a CO of 6 L/min at baseline on the electronic digital flowmeter (John C. Ernst Co, Sparta, NJ, USA) attached to the DCMS and the TAH-t monitor (CO_TAH_). The CO measured with SynCardia is denoted as CO_SYN_.

The arterial waveform from a “control” arterial line was also analyzed offline with LabChart (PowerLab^®^ pro 8, AdInstrument, Australia) and is described below (see the arterial waveform and CO_LAB_ in the Pulse Contour Analysis section).

### 2.4. Bench Control and Arterial Waveform Comparability

**Figure 1:**
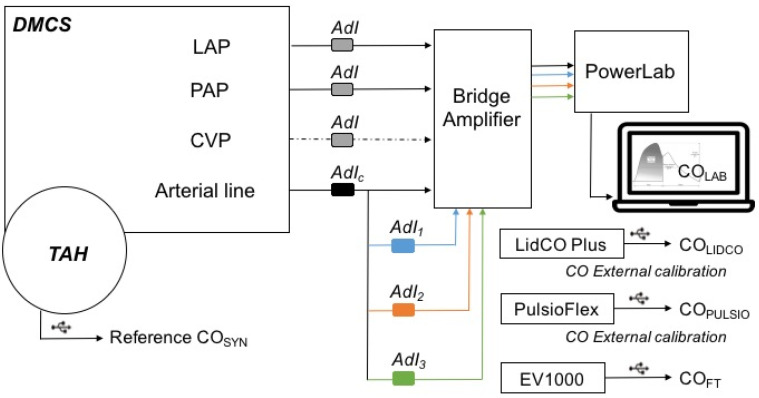
Bench control settings for comparability testing of simultaneous serial arterial waveforms.

These bench settings and the similarity of the 4 simultaneous arterial waveforms generated with the bench were at first controlled through a setting with four transducers from AdInstruments (AdI) connected with PowerLab^®^ (Figure 1). The objective was to rule out any possible effects of the serial position of a fluid-filled transducer on the several simultaneously generated arterial waveform, as shown in Figure 1. This control setting was recorded and analyzed over a 7 min test period, which represents approximately 770 cycles at 110 bpm, with pulse pressure curve analysis and comparison of systolic arterial pressure, cycle duration, dP/dt max, pulse pressure, end diastolic pressure, tau value, and estimation of the systolic area under the curve (sAUC). These parameters enabled the determination of whether comparability of the 4 simultaneous arterial waveforms was appropriate for further evaluation. A difference of less than 10% for these parameters from the 3 tested arterial waveforms (AdI 1, 2, and 3) compared with the control arterial line (AdI^CTRL^) was considered acceptable for further cross-comparisons between the devices.

### 2.5. Pulse Contour Devices

**Figure 2:**
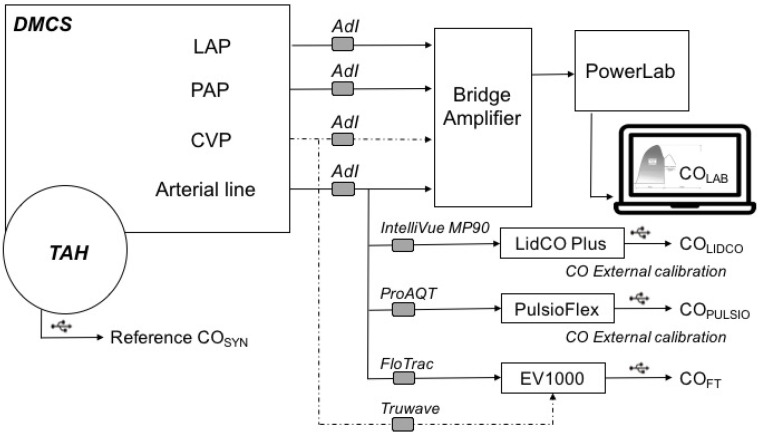
Experimental bench for pulse contour device testing.

The bench was then set up with the 3 tested pulse contour devices in order to simultaneously evaluate and compare their performance (Figure 2). 

Here, we present the current understanding of the theorethical differences between available uncalibrated devices and their algorithm used in clinical pratice.

The FloTrac™/EV1000™ system (Vigileo™, Edwards Lifesciences, Irvine, CA, USA) performs a statistical analysis of the pressure waveform at 100 Hz for 20-s intervals, capturing 2000 data points per interval. Then, the device uses an algorithm that calculates stroke volume (SV) from the pulse pressure (PP) of the arterial waveform, after correcting for the compliance and the resistance of the arterial system following the Langewouters method. Finally, this device incorporates patient height, weight, age, and mean arterial pressure for its autocalibration algorithm. This method is supported by a large database of pressure tracings recorded in hyperdynamic and vasoplegic patients [13]. The accuracy and reliability of this device are matters of debate in the literature, especially when the devices are used during major changes in arterial tone. For example, Monnet et al. found that the reliability of the third version of FloTrac ™ was much poorer for tracking changes in CO when the arterial tone was modified, as well as for changes in norepinephrine-infused doses or fluid volume infusion [14].

ProAQT/Pulsio-Flex™ (Pulsion Medical Systems, Munich, Germany), as the Langewouters method, incorporates height, weight, age, MAP, and heart rate into its autocalibration algorithm, and from these values, the CO is inferred from a company-specific and confidential analysis, which is supposed to be different from the Windkessel model [15]. However, this technique also performs pulse contour analysis of the arterial waveform 250 times per second. The accuracy of The ProAQT/Pulsio-Flex™ has also been reported to be negatively impacted by hyperdynamic states, low systemic vascular resistance (SVR), and abrupt SVR changes [16]. 

LiDCO™ Plus (LidCO™ Ltd., Cambridge, UK) uses a pulse contour algorithm based on frequency analysis studies of the arterial system [17]. It does not perform a proper analysis of pulse pressure waveforms, but it uses the physical principle of conservation of mass and pulse power in this case, with a patented PulseCO algorithm. Following calibration for compliance there is theoretically a linear relationship between net power and net flow (SV). This device with the Pulse CO algorithm performed less well than PAC-based thermodilution in patients undergoing cardiac surgery [18] or when used under very dynamic conditions such as the clamping and unclamping of the aorta in vascular surgery [19].

### 2.6. Pulse Contour Devices Settings on the Bench

The FloTrac™ sensor is associated with the EV1000™ platform (Vigileo™, v 3.0 Edwards LifeSciences, Irvine, CA, USA). proAQT™ (PulsioFlex™, Pulsion Medical system Ltd., Munich, Germany) is associated with a specific PulsioFlex™ monitor. The LiDCO™ monitor is associated with an Intellivue MP90 (Phillips, Andover, MA, USA) to record the arterial signal output, similar to usual practice. LiDCO™ Plus (LidCO Ltd., Cambridge, UK) and the PowerLab arterial lines were associated with disposable blood pressure transducers from AdInstrument (AdI) (MLT1199, BP Transducer and Cable Kit, Australia).

All tested devices were set for a male with the following measurements: height of 180 cm, weight of 80 kg, and body surface area of 2 m^2^. The CO estimates are denoted below as CO_FT_ for the Flotrac™ device used with Vigileo™, the EV1000 platform, and CO_PULSIO_, for estimates from proAQT™ attached to the PulsioFlex™ monitor or CO_LIDCO_ for LIDCO™ Plus method-related estimates.

The sampling rates differed, according to each monitor. FloTrac™, Pulsio Flex™, and LidCO™ recorded 3, 5, and 110 estimates per minute, respectively. Therefore, every CO estimate from the three tested devices were extracted through a USB port to be synchronized and averaged over a 1-min period to obtain triplicates (CO_FT_, CO_PULSIO,_ CO_LIDCO_) to compare with the CO_SYN_ and CO_LAB_ reference methods (see Figure 2). The stability of the system described above was assessed for a 50 min period.

All pulse contour devices were connected in series to the arterial pressure monitor, monitored in the Ao chamber, as described in Figure 2. The LiDCO™ Plus and PulsioFlex™ proAQT™ devices were initially calibrated for baseline CO at 6 L/min. The EV1000™ platform TruWave™ central venous pressure (CVP) transducer was connected to the RAP compartment on one side and to the Vigileo databox on the other side, for continuous monitoring of CVP.

### 2.7. Arterial Waveform and CO_LAB_ with Pulse Contour Analysis

Arterial waveform analysis was performed with Labchart™ (Labchart Pro 8, AD Instruments, Australia) to allow for the calculation of many so-called derived parameters intrinsically created by the pulse pressure profile (systolic arterial pressure, end diastolic pressure, cycle duration, pulse pressure, tau, systolic area under the curve), including estimates of left ventricular stroke volume (SV), CO, or vascular resistance [20].

Additional data recording was therefore performed using PowerLab^®^ data acquisition (PowerLab^®^ with Labchart Pro 8, AD Instruments, Australia) to enable offline pulse contour signal analysis. This “pulse contour control” with PowerLab is denoted as CO_LAB_.

Current approaches used to estimate SV from arterial pressure profiles are rooted in 1950s studies documenting that the SV is proportional to the AUC of the systolic portion of the arterial waveform [21,22]. SV=K × sysAUC × (1+ Ts/Td), where sAUC is the area under the systolic part of the arterial pressure curve (the mathematical Riemann Integral), T_sys_ and T_diast_ are the durations of systole and diastole, respectively, and K is an arbitrary constant derived from measurement of an initial SV calibrated with the reference method. In this regard, using our main pressure curve control (AdI^CTRL^), we inspected the offline AUC of the systolic portion of the arterial waveform in specific situations and, therefore, estimated a “lab pulse contour control” CO using LabChart (CO_LAB_), as CO = SV × HR. Our Ts/Td ratio was fixed during all our experiments (40% systole). The K value was calculated based on the referenced baseline SynCardia CO values for every experiment. Therefore, ${CO}_{LAB}\;=\;K\;\times \;sAUC$ × HR.

### 2.8. Protocol for System Stability Testing and Hemodynamic Challenges (HCs) on Preload, Heart Rate, and Afterload

The stability of the system with baseline parameters was first assessed for a 50 min period, before pulse contour devices were submitted to several hemodynamic challenges (HCs). All hemodynamic challenges were performed once for 10 min to obtain a stabilized hemodynamic state before returning to baseline for 5’ and then repeated 5 times for a 5 min period.

### 2.9. HC1: Preload Decrease–Hemorrhagic Shock (HS)

A steady state was observed for 10 min. Then, 3.8 L of sterile water was extracted from the CVP compartment in approximately 4 min. This fluid loss in the RAP compartment enabled an approximately 50% decrease in Syncardia cardiac output values. This period of “hemorrhagic shock” was observed for 10 min. Then, the 3.8 L were given back in the same compartment in approximately 1 min. Baseline parameters were recorded for another 5 min. This hemodynamic challenge was then repeated 5 times in a 5-min period of simulated hemorrhagic shock, while all other parameters of the bench were steady.

### 2.10. HC2: Heart Rate Increase–Tachycardia (HR 150)

The heart rate was set from the baseline, at 110 beat per min, to 150 beat per min for a 10-min period before returning to baseline. This hemodynamic challenge was repeated 5 times with a 5-min period of simulated tachycardia observed while all other parameters of the bench were steady.

### 2.11. HC3: Afterload Decrease–Low Systemic Vascular Resistance (LSVR)

Systemic vascular resistances were decreased thanks to the lower-level height of the SVR reservoir. Therefore, the responsiveness of the bellows to pressure changes in their respective chambers was voluntarily altered once for 10 min to obtain a stabilized hemodynamic state before returning to baseline for 5’, which was then repeated 5 times for a 5 min period. All other parameters of the bench were steady.

### 2.12. Statistical Analysis

In order to evaluate the degree of agreement between the devices, we performed a Bland–Altman analysis for the paired devices FT-SYN, PULSIO-SYN, and LIDCO-SYN. Bias was defined as the mean difference between the CO measurements for each paired method. The upper and lower limits of agreement were defined as ±1.96 SD of bias [23]. The reference CO measurements were Flowmeter values and CO_SYN_ and CO_LAB_ estimates offline from AdI^CTRL^ pulse contour arterial wave analysis. The error percentage and variation coefficient for each device were also assessed, as detailed in the literature (Table 1) [23]. We performed ANOVA to compare the mean CO values between the three devices with the controlled pulse contour method (CO_LAB_) and the reference method: CO_SYN_. The results are presented as the mean CO ± standard deviation. A post hoc ANOVA Dunnett’s test was used to compare groups when significance was identified, and *p* < 0.05 was considered significant. The percentage error was defined in this study as follows:

Percentage error = (1.96 × (SD of Bias between both methods))/(0.5 × (Mean tested CO method + Mean reference CO values)). All analyses were performed using GraphPad Prism 7.0 (La Jolla, CA, USA).

## 3. Results

### 3.1. Experimental Setting Control (Figure 1 and Figure 3)

**Figure 3:**
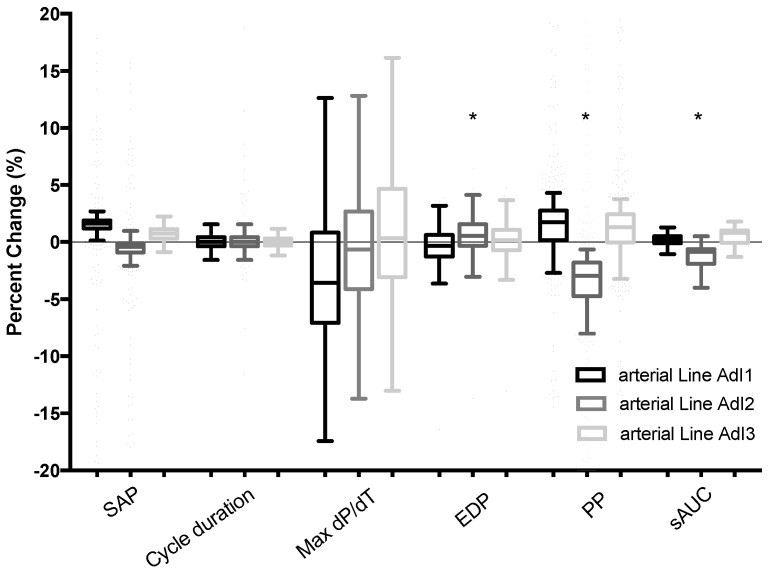
A Tukey box plot depicting the mean change to the control arterial line for systolic pressure, cycle duration, max dP/dT, end-diastolic pressure (EDP), and pulse pressure (PP) for the 3 arterial lines AdI1, 2, and 3 compared to AdI^CTRL^ (* *p* < 0.001).

The comparability of pulse signals among the four arterial lines was acceptable (<10%), with no clinically significant differences observed in systolic arterial pressure (SAP), end-diastolic pressure (EDP), cycle duration, or tau parameters. Figure 3 illustrates the three arterial lines used for LiDCO™ (AdI1), PulsioFlex™ (AdI2), and FloTrac™ (AdI3), as well as the fluid-filled transducers compared with the controlled arterial line (AdI^CTRL^) connected to PowerLab. The dP/dT_max values were 608 ± 19 (ns), 597 ± 88 (ns), and 586 ± 34 (* *p* < 0.001) compared with 605 ± 157 mmHg·s^−1^ for AdI^CTRL^. Pulse pressure (PP) values were 53.8 ± 0.4, 56.4 ± 1.8 (* *p* < 0.001), and 54.0 ± 0.3 mmHg for AdI1, AdI2, and AdI3, respectively, compared with 54.1 ± 9 mmHg for AdI^CTRL^. Similarly, the systolic area under the curve (sAUC) values were 6.54 ± 0.2, 6.63 ± 0.3 (* *p* < 0.001), and 6.51 ± 0.2 mmHg·s for AdI1, AdI2, and AdI3, respectively, compared with 6.55 ± 0.2 mmHg·s for AdI^CTRL^.

### 3.2. Stability Study

**Figure 4:**
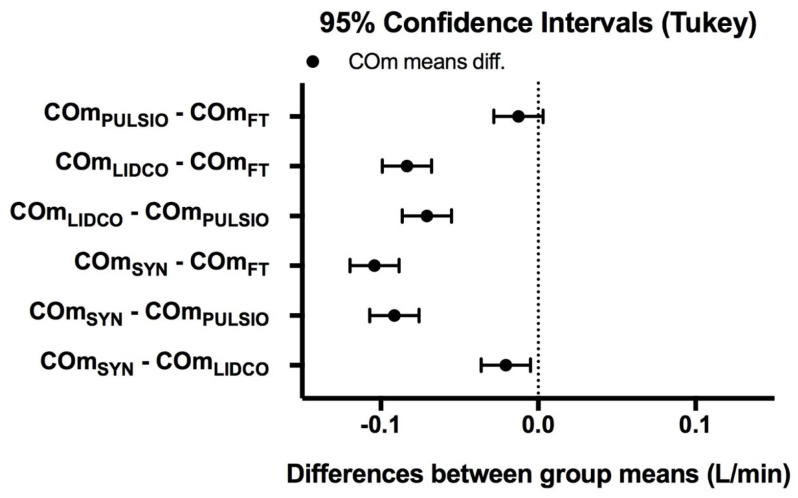
Observed difference between mean CO estimates, according to the devices at baseline.

Stability studies enabled the analysis of good congruence between the CO estimates when the bench was in steady state: CO_FT_, CO_PULSIO_, or CO_LIDCO_ were compared with Syncardia set for a CO of 6 L/min, doublechecked with a flowmeter. The mean CO estimates for Flotrac™, PulsioFlex™, LiDCO™, and SynCardia™ during this 50 min period were, respectively, 6.11 ± 0.02, 6.10 ± 0.01, 6.02 ± 0.02, and 6.00 ± 0.05 L/min. The observed difference between the mean CO estimates, according to the devices, is presented in Figure 4. The error percentages that were acceptable for the CO_FT_, CO_PULSIO_, or CO_LIDCO_ estimates were 1.69%, 1.56% and 1.61%, respectively (Table 1). The variation coefficients for the CO_FT_, CO_PULSIO_, or CO_LIDCO_ estimates were 0.31%, 0.29%, and 0.33%, respectively, compared with the CO_SYN_ variation coefficient, which was 0.85% (Table 1).

### 3.3. HC1: Preload Change–Hemorrhagic Shock (HS) (Figure 5)

**Figure 5:**
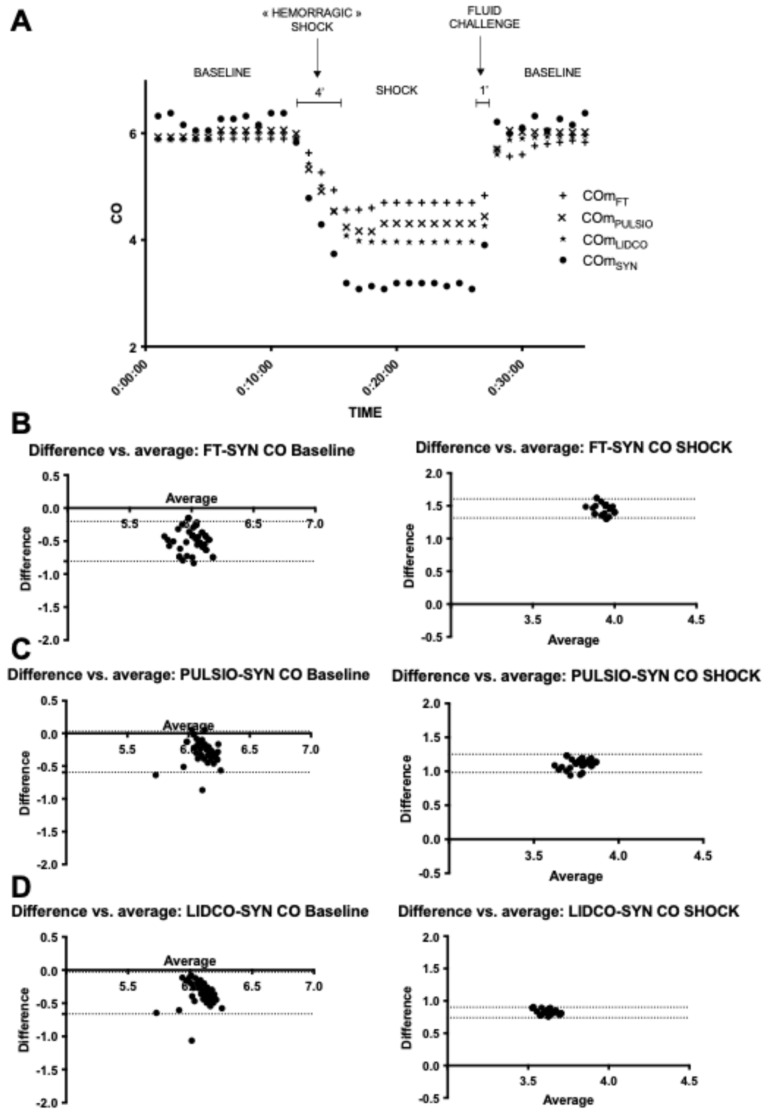
(**A**): CO estimates at baseline and during HC1: preload change–hemorrhagic shock (HS); timeline for Flotrac™ (CO_FT_), PulsioFlex™ (CO_PULSIO_), and LiDCO™ (CO_LIDCO_); (**B**): Bland et Altman plot for CO_FT_. (**C**): Bland et Altman plot for CO_PULSIO_. (**D**): Bland et Altman plot for CO_LIDCO_. The X axis represents the average of the CO estimates; the Y axis represents the difference in CO estimates for the selected devices—CO_SYN_.

At baseline, CO_PULSIO_ and CO_LIDCO_ were similar, while CO_FT_ was significantly different from the others (6.00 ± 0.12, 5.93 ± 0.12, 5.80 ± 0.11, respectively, *p* < 0.02) but within a 10% range. During HS, the mean cardiac output estimates were significantly decreased (from 19 to 49%) compared with baseline values for all devices to 4.7 ± 0.05 (*p* < 0.0001), 4.3 ± 0.07 (*p* < 0.0001), and 4.0 ± 0.05 (*p* < 0.0001) for CO_FT,_ CO_PULSIO_, and CO_LIDCO_, respectively (Figure 5A, Table 1). Figure 5 presents CO estimates at baseline and during HS, where the mean CO biases between CO_FT_, CO_PULSIO_, and CO_LIDCO_ and the reference method were, respectively, 1.5 (FT-SYN), 1.1 (PULSIO-SYN), and 0.82 (LIDCO-SYN) L/min, with limits of agreement (SD, 95% CI) of 0.07 (1.32; 1.61), 0.18 (0.98; 1.25), and 0.22, (0.74; 0.90), respectively. The percentage error for each device was 2.38%, 3.84%, and 2.63%, respectively, for the CO_FT_, CO_PULSIO_, and CO_LIDCO_ estimates when variation coefficients were 1.02%, 1.71%, and 1.2%. CO_SYN_ variation coefficient, which was 1.85% (Table 1).

### 3.4. HC2: Heart Rate Change–Tachycardia (HR150) (Figure 6 and Figure 7)

**Figure 6:**
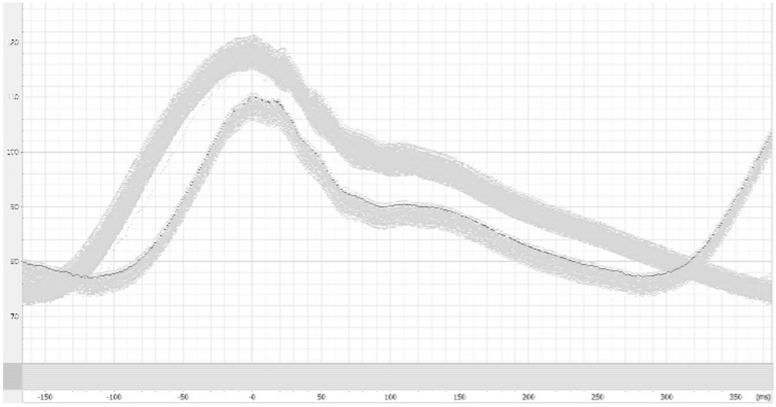
Arterial waveform morphology, when the heart rate was set from the baseline of 110 to 150 beat per min. Lower curves represent the arterial waveform of the tachycardia hemodynamic challenge.

**Figure 7:**
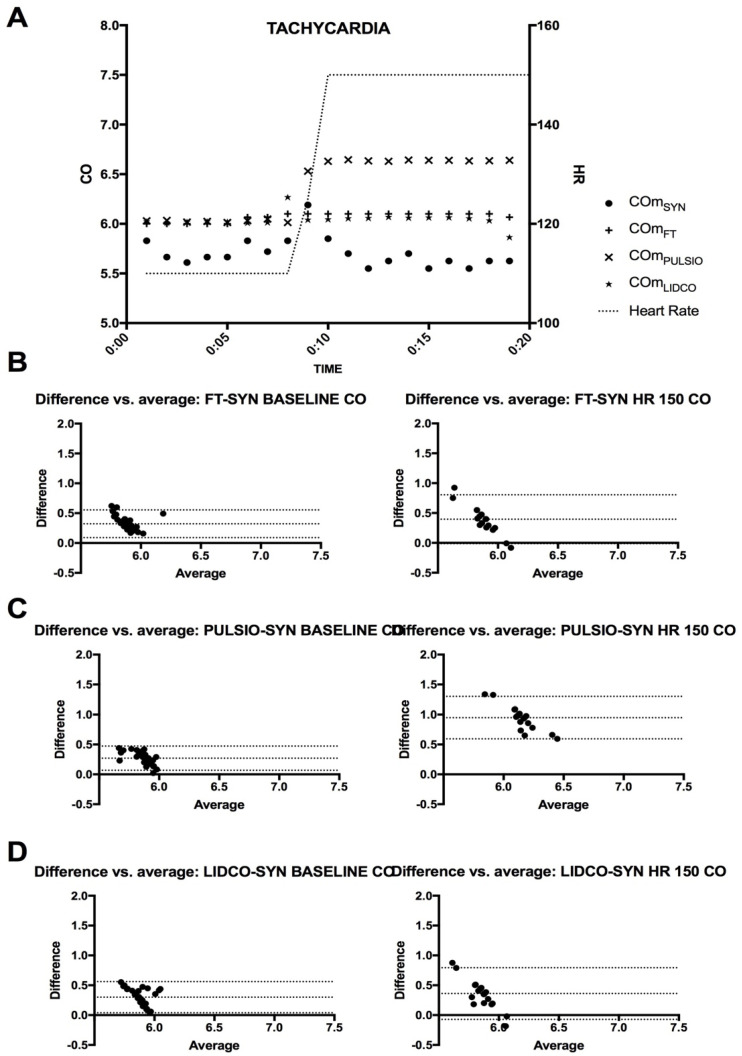
(**A**): CO estimates at baseline and during HC2: heart rate change–tachycardia (HR150); timeline for Flotrac™ (CO_FT_), PulsioFlex™ (CO_PULSIO_), and LiDCO ™ (CO_LIDCO_). (**B**): Bland et Altman plot for CO_FT_. (**C**): Bland et Altman plot for CO_PULSIO_. (**D**): Bland et Altman plot for CO_LIDCO_. The X axis represents the average of the CO estimates; the Y axis represents the difference in CO estimates for the selected devices—CO_SYN_.

At baseline, the mean CO estimates with CO_FT_, CO_PULSIO_, or CO_LIDCO_ were similar (6.06 ± 0.07, 6.00 ± 0.06, 6.03 ± 0.07 L.min^−1^, respectively, Table 2). During HR150 challenges, the mean CO estimates were significantly increased only with the Pulsio Flex device to 6.60 ± 0.06 L.min^−1^ *p* < 0.001, as presented in Figure 5A. Meanwhile, the reference CO_SYN_ values and CO_FT_ and CO_LIDCO_ estimates did not change significantly over time under HR150 compared to the baseline values (respectively, 5.6 ± 0.2 *p* = 0.29, 6.07 ± 0.03 *p* > 0.99, and 6.04 ± 0.05 L.min^−1^ *p* > 0.99). Offline CO_LAB_ estimation showed a decrease of 6% from 5.74 ± 0.14 to 5.40 ± 0.14 L.min^−1^ during tachycardia. Therefore, CO_PULSIO_ estimates were significantly higher than CO_FT_ and CO_LIDCO_ estimates (*p* < 0.0001) during HR150. Figure 7 presents CO estimates at baseline and during HR150, where the mean CO bias between CO_FT_, CO_PULSIO_, or CO_LIDCO_ and the reference method was, respectively, 0.40 (FT-SYN), 0.95 (PULSIO-SYN), and 0.36 (LIDCO-SYN) L/min, with limits of agreement (SD, 95% CI) of 0.21 (−0.01; 0.81), 0.18 (0.59; 1.30), and 0.22 (−0.07; 0.80), respectively. The percentage error for each device was 1.15%, 1.90%, and 1.66%, respectively, for CO_FT_, CO_PULSIO_, and CO_LIDCO_ estimates when the variation coefficients were 0.57%, 0.90%, and 0.82% compared with the CO_SYN_ variation coefficient, which was 3.61%. Arterial waveforms at baseline and during HR150 are presented in Figure 6.

### 3.5. HC3: Afterload Change–Low Systemic Vascular Resistance (LSVR) (Figure 8)

**Figure 8:**
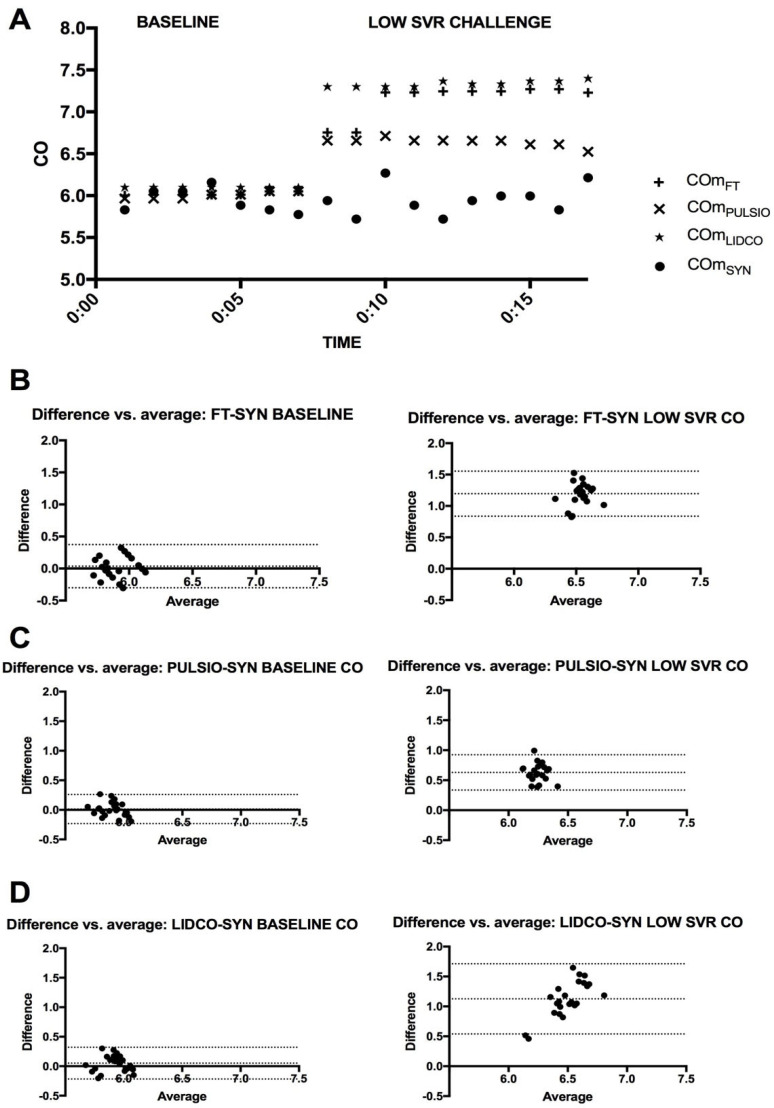
(**A**): CO estimates at baseline and during HC3: afterload change–low systemic vascular resistance (LSVR); timeline for Flotrac™ (CO_FT_), PulsioFlex™ (CO_PULSIO_), and LiDCO™ (CO_LIDCO_). (**B**): Bland–Altman Plot for CO_FT_. (**C**): Bland et Altman Plot for CO_PULSIO_. (**D**): Bland et Altman Plot for CO_LIDCO_. The X axis represents the average of the CO estimates; the Y axis represents the difference in CO estimates for the selected devices—CO_SYN_.

At baseline, the CO_FT_, CO_PULSIO_, and CO_LIDCO_ values were 5.93 ± 0.16, 5.91 ± 0.10, and 5.95 ± 0.13 L.min^−1^, respectively. The mean CO estimates for Flotrac™, Pulsio flex™, and LiDCO™ during LSVR were significantly increased from 5.93 ± 0.16 to 7.06 ± 0.18, 5.91 ± 0.10 to 6.57 ± 0.15, and 5.95 ± 0.13 to 7.13 ± 0.30 L.min^−1^, respectively (see Figure 8A). Conversely, the CO_SYN_ values did not change significantly (5.90 ± 0.13 at baseline vs. 5.94 ± 0.11, *p* = 0.26). The CO_SYN_ estimates during LSVR were significantly lower than the CO_FT_ and CO_LIDCO_ estimates (*p* < 0.0001). CO_LAB_ enabled a 7.3% increase from 5.90 ± 0.13 to 6.33 ± 0.13 L.min^−1^ during the LSVR challenge. Figure 8 presents the CO estimates at baseline and during low systemic vascular resistance. During LSVR, the mean CO bias between CO_FT_, CO_PULSIO_, or CO_LIDCO_ and the reference method was, respectively, 1.20 (FT-SYN), 0.63 (PULSIO-SYN), and 1.13 (LIDCO-SYN) L/min, with limits of agreement (SD, 95% CI) of 0.18, (0.84; 1.55), 0.15, (0.34; 0.92), and 0.30, (0.54; 1.71), respectively. The percentage errors for each device were 8.62%, 3.01%, and 3.92%, respectively, for the CO_FT_, CO_PULSIO_, and CO_LIDCO_ estimates when the variation coefficients were 4.03%, 1.46%, and 1.83% compared with the CO_SYN_ variation coefficient, which was 1.83%.

## 4. Discussion

The present study revealed that a Donovan mock circulatory system (DMCS) paired with a total artificial heart (TAH-t™) provides a robust experimental hemodynamic simulation bench for evaluating pulse contour devices under controlled simultaneous conditions. We demonstrated that our innovative bench model is stable, accurate, and reliable in generating controlled pulse pressure waveforms. This setup allows for the manipulation of hemodynamic variables—such as the preload, heart rate, and systemic vascular resistance—enabling a comprehensive assessment of device performance under various physiological hemodynamic challenges. Each pulse contour device employs different algorithms and methodologies to estimate CO, raising questions about their relative accuracy and reliability. Despite their common origins and intended purposes, pulse contour devices that rely on arterial waveform analysis display substantial heterogeneity. This experimental study’s design, incorporating Bland–Altman analysis, is appropriate for determining the degree of agreement between the pulse contour devices and the reference method. We found acceptable agreement among the devices under baseline conditions, with percentage errors and variation coefficients remaining within acceptable clinical limits. However, the hemodynamic challenges posed significant variability in device performance.

During HC1, preload change–hemorrhagic shock simulation, all devices demonstrated a marked decrease in CO estimates, yet differences among them became evident. Such findings suggest that while all devices can track changes, the degree to which they do may depend on the nature of the hemodynamic disturbance. This study highlights that, during HC2, heart rate change–tachycardia simulation with a 150 Bpm heart rate, only the PulsioFlex™ device significantly increased its CO estimate. In contrast, FloTrac™ and LiDCO™ recorded CO measurements similar to baseline, while CO_SYN_ remained stable and CO_LAB_ decreased by approximately 6%. This divergence likely reflects the limitation of contour-based reference estimation with an elevated heart rate, where shorter cycle durations and altered pulse pressure morphology can distort waveform-based calculations. The mechanical output confirms that true cardiac output did not vary significantly, supporting CO_SYN_ as the more physiologically valid reference under these conditions.

In the context of HC3, afterload change–low systemic vascular resistance simulation, all three tested devices showed CO overestimation. However, we observed a closer association in CO estimates between CO_LAB_ and CO_PULSIO_ compared with the other devices (CO_LIDCO_ or CO_FT_). In particular, the CO_PULSIO_ and CO_LAB_ pulse contour method measurements drifted less than other devices regarding the CO_SYN_ reference value. This difference between CO_LAB_ and CO_PULSIO_ (small drift) on one side and CO_LIDCO_ or CO_FT_ (significant overestimation) on the other side during LSVR could be explained by both CO_LAB_ and CO_PULSIO_ sharing the area under the curve parameter in their mathematical equations. This result underscores the importance of understanding individual device strengths and limitations in varying clinical scenarios.

These findings, reporting a variability in performance among the devices in our experimental hemodynamic challenges simulations, suggest that some devices may be more suited to specific clinical scenarios and highlight the critical need for clinicians to choose the right monitoring device based on the patient’s hemodynamic state. The clinical implications of this study are significant. Accurate CO monitoring is essential for guiding fluid management, assessing cardiac function, and informing therapeutic interventions in critically ill patients. Our results are consistent with the literature; several authors have emphasized the limitations of pulse contour devices’ performance under various clinical conditions [8,16,18,19].

Several clinical studies have reported significant variability in the performance of uncalibrated pulse contour algorithms, compared to a reference cardiac output measurement technique (thermodilution or echocardiography) [9]. Several parameters may limit the accuracy and observed performance of pulse contour algorithms and commercially available devices. Aortic impedance, which is influenced by the arterial cross-sectional area and compliance, represents the opposition to pulsatile aortic inflow. Compliance represents the resistance of the aorta to volume increases (dV/dP), which exhibit non-linear behavior (roughly exponential, with a substantial decrease when arterial pressure increases significantly). Langewouters studied this non-linear behavior in vitro (in fresh cadavers) and mathematically described how compliance varies with age, gender, and height [15]. The cross-sectional area is also dependent on age, sex, and height, but individual patients may deviate from Langewouters’ population by 30%. Systemic vascular resistance (arterial tone) is the opposition of systemic vascular beds to blood drainage. It is a dynamic factor that depends on many other factors, including circulatory filling, sympathetic tone, temperature, and vasoactive drugs. To further complicate matters, the arterial pressure wave is composed not only of the incident wave but also of reflected waves, a source of interference that must be accounted for [24]. This phenomenon is most important when invasive arterial catheters are sited peripherally, as in the present setting, in which the arterial pressure wave is distorted.

While our study’s findings are promising, there are limitations worth noting. The artificial nature of this innovative hemodynamic bench may not fully replicate the complex interactions present in human physiology. Interestingly, CO_SYN_ values from our reference method did not differ between the baseline and the afterload decrease. The present finding may be explained by the fact that the change in systemic vascular resistance in the Mock circulatory system does not affect TAH-t outflow, as the machine is able to adapt to a resistance modification without changing its stroke volume cycling (linear cardiovascular coupling) with no changes in systolic and diastolic time. Indeed, in our model, cardiovascular coupling is not physiological, as there are no perpetual nervous and humoral beat-to-beat adaptations of capillaries or microcirculation to stroke volumes changes. Moreover, cardiovascular coupling is not under humoral control as in humans (epinephrine, norepinephrine, or NO), and in this regard, the authors cannot be certain that existing algorithms are designed to act in response to permanent physiological retro-control and feedback.

Future studies should aim to validate these findings in more complex simulated hemodynamic challenges, as well as in controlled in clinical settings with real patients to ascertain the external validity of the results.

Furthermore, our study did not explore the impact of patient-specific factors such as age, co-morbidities, and baseline hemodynamics, which could influence the performance of the device. Future research should consider improving the capability of the experimental rig to reproduce and simulate a wider range of hemodynamic states, closer to real patient conditions. As hospitals strive to select suitable, high-performance tools and adopt sustainable practices, assessing the overall performance, cost-effectiveness and life-cycle costs of these pulse contour devices could guide future purchasing decisions.

## 5. Conclusions

This study highlights the efficacy of an innovative hemodynamic simulation bench as a reliable experimental model for evaluating the accuracy of various continuous cardiac output monitoring devices. Developing the present innovative experimental pulsatile bench to potentially further evaluate several simultaneous uncalibrated pulse contour devices and to submit their pulse contour algorithm to in vitro hemodynamic challenges is of great interest. The ability to test the performance of continuous cardiac output monitoring devices using a simulated circulatory system, with accurate and reproducible flow measurement, provides valuable insight into their relative accuracy and reliability, while preserving the safety of our patients. Our study underscores the complexity of hemodynamic monitoring in critically ill patients and the necessity for tailored device selection based on specific clinical scenarios.

Ultimately, this research emphasizes the need for continued exploration of these technologies to ensure optimal patient outcomes in critical care settings. By improving our understanding of pulse contour devices, clinicians can make more informed decisions about cardiovascular monitoring strategies, potentially enhancing the management of critically ill patients. Future research should focus on translating these findings into clinical practice, examining long-term impacts on patient outcomes and guiding best practices in hemodynamic monitoring.

## Figures and Tables

**Table 1:** Pulse contour device’s cardiac output estimate, error percentage, and variation coefficient in steady state and HC1: preload change–hemorrhagic shock (HS).

Pulse Contour Devices	Steady State	EP (%)	VC (%)	Baseline	HC1	EP (%)	VC (%)
CO_FT_	6.11 ± 0.02	1.69	0.31	6.00 ± 0.12	4.7 ± 0.05	2.38	1.02
CO_PULSIO_	6.10 ± 0.01	1.56	0.29	5.93 ± 0.12	4.3 ± 0.07	3.84	1.71
CO_LIDCO_	6.02 ± 0.02	1.61	0.33	5.80 ± 0.11	4.0 ± 0.05	2.63	1.20
CO_SYN_	6.00 ± 0.05	-	0.85	6.30 ± 0.13	3.2 ± 0.06	-	1.85

Cardiac output estimates are presented as CO ± SD (L/min); EP, error percentage; VC, variation coefficient. HC1: preload change–hemorrhagic shock (HS).

**Table 2:** Pulse contour device’s cardiac output estimate, error percentage, and variation coefficient, upon several hemodynamic challenges.

Pulse Contour Devices	Baseline	HC2	EP (%)	VC (%)	Baseline	HC3	EP (%)	VC (%)
CO_FT_	6.06 ± 0.07	6.07 ± 0.03	1.15	0.57	5.93 ± 0.16	7.06 ± 0.18	8.62	4.03
CO_PULSIO_	6.00 ± 0.06	6.60 ± 0.06	1.90	0.90	5.91 ± 0.10	6.57 ± 0.15	3.01	1.46
CO_LIDCO_	6.03 ± 0.07	6.04 ± 0.05	1.66	0.82	5.95 ± 0.13	7.13 ± 0.30	3.92	1.83
CO_SYN_	5.73 ± 0.12	5.60 ± 0.20	-	3.61	5.90 ± 0.13	5.94 ± 0.11	-	1.83

Cardiac output estimate is presented as CO ± SD (L/min); EP, error percentage; VC, variation coefficient. HC2: heart rate change–tachycardia (HR150); HC3: afterload change–low systemic vascular resistance (LSVR).

## Data Availability

The original contributions presented in the study are included in the article, further inquiries can be directed to the corresponding authors.

## Supplementary Materials

The following supporting information can be downloaded at: https://www.mdpi.com/article/10.3390/jcm14228030/s1, Description of the mock pulsatile circulatory system; Figure S1: Picture from Geneva hemodynamic research group lab; Video Settings Donovan Mock Circulatory System with SynCardia TAH with settings video links: *Film 1*, *Film 2*.
