# Supplementary material for: Simultaneous Evaluation of Pulse Contour Devices Using an Innovative Hemodynamic Simulation Bench
Source: J Clin Med. 2025 Nov 12;14(22):8030. doi: 10.3390/jcm14228030 (PMC12653771; doi:10.3390/jcm14228030)
Supplement: Supplementary file 1 [file jcm-14-08030-s001.zip › jcm-3895547-supplementary.pdf]

Journal of Clinical Medicine

Simultaneous Evaluation of Pulse Contour Devices Using an Innovative Hemodynamic Simulation Bench

Paul Samuel Abraham [1,2,*], Bernardo Bollen Pinto [2,3], Raphael Giraud [3], Thomas Millien [3], Sylvain Thuaudet [4] and Karim Bendjelid [2,3,*]

[1] Anaesthesiology Department, Lausanne University Hospital, 1011 Lausanne, Switzerland
[2] Faculty of Medicine, University of Geneva, 1211 Geneva, Switzerland; bernardo.bollenpinto@hug.ch
[3] Department of Anaesthesiology, Pharmacology, Intensive Care and Emergency Medicine, Geneva University Hospital, 1211 Geneva, Switzerland; raphael.giraud@hug.ch (R.G.); thomas.millien@hug.ch (T.M.)
[4] S.T. Consulting, 14480 Le Fresne-Camilly, France; st@stc-consulting.fr
* Correspondence: paul.abraham@chuv.ch (P.S.A.); karim.bendjelid@hug.ch (K.B.); Tel.: +41-21-314-20-07 (P.S.A.)

SUPPLEMENTARY MATERIALS

- **Figure S1: Picture Settings from Geneva hemodynamic research group lab**

- **Video- Settings from Geneva hemodynamic research group lab.**

Donovan Mock Circulatory System with SynCardia TAH with
Settings video links: Film 1 , Film 2

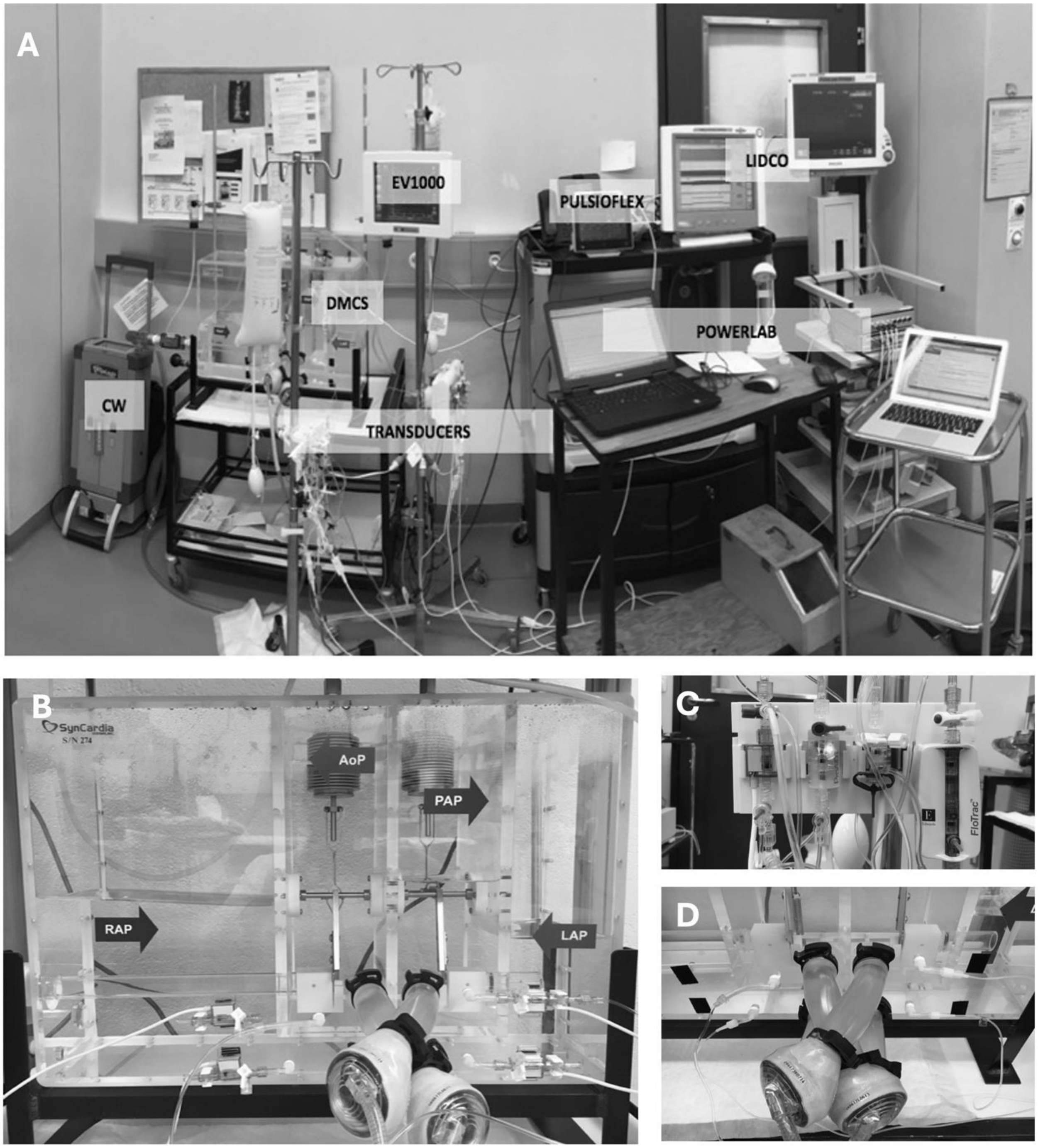

Figure S1. Haemodynamic Simulation Bench. A Settings from Geneva hemodynamic research group lab. B Donovan 4 chambers Mock Circulatoy system. C Pulse contour Transduceur. D Total Artificial heart Syncardia™ Artificial ventricles. **CW : CardioWest Total Artificial Heart Driver system, DMCS Donovan Mock Circulatoy system.**

The mock pulsatile circulatory system (Figure S1)

This Donovan Mock Circulatory System DMCS) consists of a clear plastic tank divided into four chambers to simulate the four representative pressure chambers of the heart (i.e., right atrial pressure [RAP], pulmonary artery pressure [PAP], left atrial pressure [LAP] and aortic pressure [AoP]). The AoP and RAP chambers and the PAP and LAP chambers were connected

 https://doi.org/10.3390/jcm14228030

through flow tubes. The temporary TAH-t was attached to the appropriate chambers of the mock circulation with inflow and outflow conduits (Figure S1,B). A toggle valve, which is used to preset the appropriate compliance level for each chamber, was located at the top and rear of each chamber. The AoP, PAP and LAP chamber toggles remained closed. An RAP chamber toggle was kept open. These four tanks contained different water volumes to obtain the desired and adequate pressure in each chamber. The total volume was 5 US gallons (18.9 liters). The residual air volume, present in each reservoir, allowed for pressure control changes. The flow of volume from one reservoir to another resulted in diminished pressure in the original reservoir and increased pressure in the end reservoir (Boyle's Law). These four chambers were connected among themselves and internally with different tubes to reproduce elastic systemic and pulmonary circulations. These tanks were also connected externally through four polyvinyl chloride tubes to a biventricular pump (TAH-t) (SynCardia™ Systems Inc., Tucson, AZ) which produced a pulsatile heart, driven by pneumatic energy (Figure S1,D). Between the tanks representing the aorta and the right atrium on the one hand and the pulmonary artery and the left atrium on the other hand was a system that recreated the systemic and pulmonary vascular resistances and compliances (modulus)(1). The AoP and PAP chambers contained bellows, which were attached to gate valves. The gate valves provided resistance to the flow of water from the AoP to the RAP and from the PAP to the LAP chambers, thereby simulating systemic and pulmonary vascular resistance, respectively. The bellows were filled with water, and the water column in the bellows was connected to the moveable reservoirs with clear plastic tubes. By changing the height of the reservoirs, the bellows' responsiveness to pressure changes in their respective chambers could be altered, which then altered the closure rate of the gate valves, allowing for manipulation of systemic and pulmonary vascular resistance. Raising the reservoir increases the resistance and pressure in the chamber. Lowering the reservoir decreased the resistance and pressure in the chamber. The reservoirs were labelled SVR for systemic vascular resistance and PVR for pulmonary vascular resistance. The mock system was associated with an electronic digital flowmeter (John C. Ernst Co, Sparta, NJ, USA) placed between the AoP and RAP chambers.

The artificial ventricles

In this experimental bench, the ventricular chambers were simulated using two 70 ml pneumatically driven, pulsatile ventricles from a TAH-t (SynCardia™ Systems Inc., Tucson, AZ). Each of the TAH-t ventricles had a rigid spherical outer housing that supported a blood-contacting diaphragm, two inner diaphragms, and an air-contacting diaphragm, all fabricated together from segmented polyurethane. Artificial ventricles from the TAH-t are powered by pneumatic energy, and they were controlled in this experimental model using an external pneumatic console (i.e., dual drive console from SynCardia™) (Figure S1,A (CW)). Each artificial ventricle consisted of two compartments separated by a polyurethane membrane. One of these compartments was the ejection chamber of the ventricle, with the "blood" content, and the other is a chamber with air content in which the pressure was controlled by the pneumatic console. Mobilizing this polyurethane membrane enabled the ejection chamber to be completely empty, with the flow direction being controlled by two Medtronic Hall mechanical valves (Medtronic Inc., Minneapolis, MN, USA) simulating the inflow and outflow valves (mitral/aortic and tricuspid/ pulmonary) of each ventricle. The maximum output volume was that obtained from the output chamber of these pneumatic ventricles: 70 ml. The present bench testing model enables the user control the beat rate, percentage of systole, and left and right driving pressure. The CO_{SYN} value is based on the volume of airflow

out of the drivelines, as well as the trend plots of the left-sided and right-sided CO, which were continuously displayed as separate ventricular fill volumes.

Pulsatile signal output.

Pressure in all 4 tanks was continuously measured and displayed in real time on an adjacent screen (PowerLab® pro 8, AdInstrument, Sidney, Australia). Each chamber disposed of a pressure line that allowed monitoring of the maximum, minimum and mean pressure in every tank. Therefore, a pressure transducer connected to these chambers provided artificial right atrial pressure (RAP), pulmonary artery pressure (PAP), left atrial pressure (LAP) and aortic pressure (AoP) in mmHg. The observed values were similar to the physiological conditions of the chosen dual drive console settings described

earlier. After calibrating and zeroing, the pulsatile signal quality enabled observation of systolic and diastolic pressure, with the dicrotic notch. The quality of the arterial signal through the fluid-filled catheter associated with the flushing bag pressure at 300 mmHg was controlled with a fast flushing square wave test before every data recording to exclude an over- or under-damped arterial line waveform (2).

References

1. Fukumitsu, M.; Kawada, T.; Shimizu, S.; Turner, M.J.; Uemura, K.; Sugimachi, M. Development of a servo pump system for in vivo loading of pathological pulmonary artery impedance on the right ventricle of normal rats. *Am. J. Physiol. Heart. Circ. Physiol.* **2016**, 310, H973–H983.
2. Kleinman, B.; Powell, S.; Gardner, R.M. Equivalence of fast flush and square wave testing of blood pressure monitoring systems. *J. Clin. Monit.* **1996**, *12*, 149–154.
